# Melaminium 2,4,6-trihydroxy­benzoate dihydrate

**DOI:** 10.1107/S1600536809031055

**Published:** 2009-08-12

**Authors:** Timothy J Prior, Osman Goch, Rebecca L Kift

**Affiliations:** a Department of Chemistry, University of Hull, Kingston upon Hull HU6 7RX, England

## Abstract

In the title compound, C_3_H_7_N_6_^+^·C_7_H_5_O_5_^−^·2H_2_O, the melaminium and benzoate ions are approximately planar (r.m.s. deviation of the non-hydrogen atoms is 0.093 Å) and there is a strong *C*_2_^2^(8) hydrogen-bonding embrace between them. The centre of symmetry generates a second acid–base pair which is bound to the first by a *C*_2_^2^(8) (N—H⋯N) embrace common between melamine mol­ecules in similar compounds. Further extensive hydrogen bonding assembles the components into a three-dimensional hydrogen-bonded network.

## Related literature

For 2,4,6-trihydroxy­benzoic acid and some of its compounds, see: Jankowski *et al.* (2007 ▶). For compounds of melamine with aromatic acids, see: Zhang & Chen (2005 ▶); Perpétuo & Janczak (2005 ▶); Zhang *et al.* (2004 ▶); Karle *et al.* (2003 ▶); Janczak & Perpétuo (2001 ▶). For a description of the Cambridge Crystallographic Database, see: Allen (2002 ▶). For a structure related to the title compound with C—H⋯O inter­actions with a homomeric 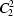
(8) motif, see: Bouvet *et al.* (2007 ▶).
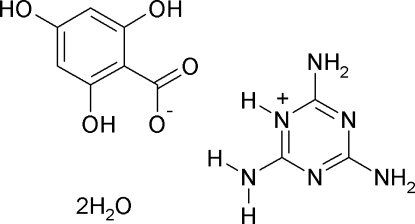


## Experimental

### 

#### Crystal data

C_3_H_7_N_6_^+^·C_7_H_5_O_5_^−^·2H_2_O
*M* *_r_* = 332.29
Monoclinic, 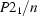

*a* = 6.9914 (6) Å
*b* = 11.7105 (14) Å
*c* = 17.1784 (14) Å
β = 93.247 (7)°
*V* = 1404.2 (2) Å^3^
*Z* = 4
Mo *K*α radiation
μ = 0.13 mm^−1^
*T* = 150 K
0.55 × 0.30 × 0.24 mm

#### Data collection

Stoe IPDS2 diffractometer
Absorption correction: analytical (*X-RED*; Stoe & Cie, 2002 ▶) *T*_min_ = 0.945, *T*_max_ = 0.973
17706 measured reflections
6005 independent reflections
3332 reflections with *I* > 2σ(*I*)
*R*_int_ = 0.047

#### Refinement

*R*[*F* ^2^ > 2σ(*F* ^2^)] = 0.042
*wR*(*F* ^2^) = 0.127
*S* = 0.85
6005 reflections
272 parameters
31 restraints
All H-atom parameters refined
Δρ_max_ = 0.46 e Å^−3^
Δρ_min_ = −0.36 e Å^−3^

### 

Data collection: *X-AREA* (Stoe & Cie, 2002 ▶); cell refinement: *X-AREA*; data reduction: *X-RED* (Stoe & Cie, 2002 ▶); program(s) used to solve structure: *SHELXS97* (Sheldrick, 2008 ▶); program(s) used to refine structure: *SHELXL97* (Sheldrick, 2008 ▶); molecular graphics: *ORTEP-3* (Farrugia, 1997 ▶); software used to prepare material for publication: *SHELXL97*.

## Supplementary Material

Crystal structure: contains datablocks global, I. DOI: 10.1107/S1600536809031055/zl2230sup1.cif

Structure factors: contains datablocks I. DOI: 10.1107/S1600536809031055/zl2230Isup2.hkl

Additional supplementary materials:  crystallographic information; 3D view; checkCIF report

## Figures and Tables

**Table 1:** Hydrogen-bond geometry (Å, °)

*D*—H⋯*A*	*D*—H	H⋯*A*	*D*⋯*A*	*D*—H⋯*A*
O1*W*—H1*B*⋯O2	0.88 (2)	1.96 (2)	2.8015 (15)	160 (2)
O1*W*—H1*A*⋯O3^i^	0.887 (19)	2.04 (2)	2.8329 (13)	148 (2)
O2*W*—H2*B*⋯O5	0.897 (19)	2.01 (2)	2.8835 (16)	166 (2)
O2*W*—H2*A*⋯O1*W*^ii^	0.906 (19)	1.903 (19)	2.8050 (15)	173 (2)
N1—H1⋯O1	0.908 (16)	1.869 (16)	2.7729 (13)	173.9 (19)
N4—H4*A*⋯O2	0.942 (17)	1.886 (17)	2.8245 (14)	174 (2)
N4—H4*B*⋯O5^iii^	0.890 (14)	2.214 (16)	3.0049 (14)	147.9 (16)
N5—H5*A*⋯O2*W*^iv^	0.902 (15)	2.145 (17)	2.8319 (15)	132.2 (15)
N5—H5*B*⋯N2^v^	0.917 (15)	2.017 (15)	2.9339 (15)	179.0 (17)
N6—H6*A*⋯O3^vi^	0.902 (15)	2.334 (16)	3.1215 (14)	145.9 (16)
N6—H6*B*⋯O2*W*^vii^	0.905 (15)	2.013 (16)	2.9096 (15)	170.2 (17)
O3—H3⋯O1	0.928 (19)	1.64 (2)	2.5235 (12)	156 (2)
O4—H4*C*⋯O1*W*^viii^	0.880 (18)	1.858 (18)	2.7284 (13)	170 (2)
O5—H5⋯O2	0.921 (19)	1.70 (2)	2.5461 (13)	151 (2)
C6—H6⋯O4^ix^	0.975 (17)	2.582 (17)	3.5529 (15)	173.9 (15)

Symmetry codes: (i) 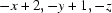; (ii) 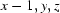; (iii) 
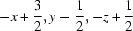
; (iv) 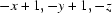; (v) 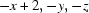; (vi) 
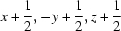
; (vii) 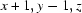; (viii) 
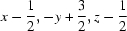
; (ix) 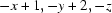.

**Fig. 1.**
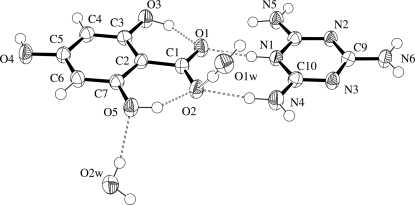
: ORTEP plot of the asymmetric unit of the title compound. Atoms are drawn as 70% thermal ellipsoids. Dashed lines represent hydrogen bonds.

**Fig. 2.**
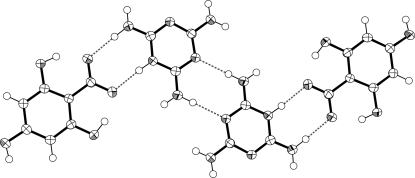
: Repeat unit of the chains present in the title compound.

**Fig. 3.**
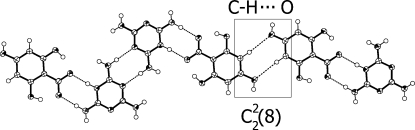
: Part of one of the infinite chains is illustrated. The weak dimeric C—H···O (phenol) interaction joining the links is highlighted.

**Fig. 4.**
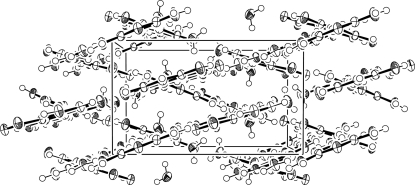
: View of the structure down [001]. Stacks of chains running parallel to [120] and [120] are visible.

## supplementary crystallographic information

### Comment

Melamine (2,4,6-triamino-*s*-triazine, C_3_H_6_N_6_) has been widely studied for its potential in the formation of extended hydrogen-bonded solids. For example, crystals of melamine with the following aromatic acids have been reported: benzoic acid (Perpétuo & Janczak, 2005); phthalic acid (Janczak & Perpétuo, 2001); terephthalic acid (Zhang *et al.*, 2004, and Zhang & Chen, 2005); mellitic acid (Karle *et al.*, 2003). The structure of 2,4,6-trihydroxybenzoic acid and those of some co-crystals of this acid have been reported previously by Jankowski **et al.** (2007). Here we report a co-crystal of melamine and 2,4,6-trihydroxybenzoic acid obtained from aqueous solution.

The title compound crystallizes in the centrosymmetric space group *P*2_1_/*n* with four formula units in the unit cell. The 2,4,6-trihydroxybenzoic acid molecule is deprotonated at the carboxylic acid function. One of the nitrogen atoms of the triazine ring of melamine is protonated. This acid-base pair forms a complementary C_2_^2^(8) embrace illustrated in Figure 1. Details of the hydrogen bonding within this structure are given in Table 1. A second acid-base pair is generated by the inversion centre. This forms a pair of strong hydrogen bonds to the first through the two melaminium ions. This melamine-melamine C_2_^2^(8) embrace is observed in many other compounds involving melamine. This four molecule unit (illustrated in Figure 2) can be thought of as the repeat unit in an infinite chain. These links are held together by weaker, non-classical C—H···O hydrogen bonds between the C6-(H6) and the hydroxyl group (O4) of another acid unit. This is illustrated in Figure 3. The C···O distance here is 3.5529 (15) Å and the H···O distance is 2.5812 (17) Å. A brief review of about 350 structures with a similar C—H···O (aromatic hydroxyl) homomeric C_2_^2^(8) embrace in the Cambridge Structural Database (Allen, 2002) reveals that the distances and geometry displayed here are in good agreement with those previously reported. One similar example is reported by Bouvet *et al.* (2007).

Infinite chains formed from this repeat unit are arranged in stacks. The vertical separation between chains is 3.3496 (5) Å. These stacks of chains are arranged along the *c* axis. The chains within adjacent stacks are alternately parallel to the [120] and [120] directions. The angle between chains parallel to [120] and [120] is 33.242 (8) °. A view of part of the structure down the crystallographic *c* axis is shown in Figure 4. Between these stacks a large number of classical hydrogen bonds are formed. These are reinforced by the presence of the two water molecules. Full details are given in Table 1.

### Experimental

A solution of melamine and 2,4,6-trihydroxybenzoic acid (0.012 mol dm^-3^ in each component) was prepared in deionized water. 5 mL portions of this solution were allowed to evaporate at room temperature in air from suitably sized vials. After a period of approximately two weeks, good sized colourless crystals were obtained.

### Refinement

The data were of sufficient quality to allow identification of all the hydrogen atoms within a difference Fourier map once the heavier atoms had been located. The positions and displacement parameters of the hydrogen atoms were refined independently subject to soft restraints that chemically equivalent bonds should have similar lengths.

### Crystal data

**Table:**

C_3_H_7_N_6_^+^·C_7_H_5_O_5_^−^·2H_2_O	*F*(000) = 696
*M**_r_* = 332.29	*D*_x_ = 1.572 Mg m^−^^3^
Monoclinic, *P*2_1_/*n*	Mo *K*α radiation, λ = 0.71073 Å
Hall symbol: -P 2yn	Cell parameters from 10461 reflections
*a* = 6.9914 (6) Å	θ = 3.0–34.7°
*b* = 11.7105 (14) Å	µ = 0.13 mm^−^^1^
*c* = 17.1784 (14) Å	*T* = 150 K
β = 93.247 (7)°	Block, colourless
*V* = 1404.2 (2) Å^3^	0.55 × 0.30 × 0.24 mm
*Z* = 4	

### Data collection

**Table:**

Stoe IPDS2 diffractometer	6005 independent reflections
Radiation source: fine-focus sealed tube	3332 reflections with *I* > 2σ(*I*)
graphite	*R*_int_ = 0.047
Detector resolution: 6.67 pixels mm^-1^	θ_max_ = 34.8°, θ_min_ = 2.9°
ω scans	*h* = −11→9
Absorption correction: analytical (*X-RED*; Stoe & Cie, 2002)	*k* = −18→16
*T*_min_ = 0.945, *T*_max_ = 0.973	*l* = −27→27
17706 measured reflections	

### Refinement

**Table:**

Refinement on *F*^2^	Primary atom site location: structure-invariant direct methods
Least-squares matrix: full	Secondary atom site location: difference Fourier map
*R*[*F*^2^ > 2σ(*F*^2^)] = 0.042	Hydrogen site location: difference Fourier map
*wR*(*F*^2^) = 0.127	All H-atom parameters refined
*S* = 0.85	*w* = 1/[σ^2^(*F*_o_^2^) + (0.0793*P*)^2^] where *P* = (*F*_o_^2^ + 2*F*_c_^2^)/3
6005 reflections	(Δ/σ)_max_ < 0.001
272 parameters	Δρ_max_ = 0.46 e Å^−^^3^
31 restraints	Δρ_min_ = −0.36 e Å^−^^3^

### Special details

**Table:**

Geometry. All e.s.d.'s (except the e.s.d. in the dihedral angle between two l.s. planes) are estimated using the full covariance matrix. The cell e.s.d.'s are taken into account individually in the estimation of e.s.d.'s in distances, angles and torsion angles; correlations between e.s.d.'s in cell parameters are only used when they are defined by crystal symmetry. An approximate (isotropic) treatment of cell e.s.d.'s is used for estimating e.s.d.'s involving l.s. planes.
Refinement. Refinement of *F*^2^ against ALL reflections. The weighted *R*-factor *wR* and goodness of fit *S* are based on *F*^2^, conventional *R*-factors *R* are based on *F*, with *F* set to zero for negative *F*^2^. The threshold expression of *F*^2^ > σ(*F*^2^) is used only for calculating *R*-factors(gt) *etc*. and is not relevant to the choice of reflections for refinement. *R*-factors based on *F*^2^ are statistically about twice as large as those based on *F*, and *R*- factors based on ALL data will be even larger.

### Fractional atomic coordinates and isotropic or equivalent isotropic displacement parameters (Å^2^)

**Table:**

	*x*	*y*	*z*	*U*_iso_*/*U*_eq_	
C1	0.73313 (18)	0.52661 (10)	0.05113 (6)	0.0196 (2)	
C2	0.68970 (18)	0.63258 (9)	0.00699 (6)	0.0183 (2)	
C3	0.69836 (18)	0.63594 (10)	−0.07492 (6)	0.0194 (2)	
H3	0.776 (3)	0.4883 (18)	−0.0746 (12)	0.056 (6)*	
C4	0.65003 (19)	0.73346 (10)	−0.11738 (6)	0.0211 (2)	
H4	0.660 (2)	0.7325 (14)	−0.1742 (9)	0.023 (4)*	
C5	0.59022 (19)	0.83049 (10)	−0.07820 (6)	0.0210 (2)	
C6	0.5819 (2)	0.83144 (10)	0.00264 (6)	0.0216 (2)	
H6	0.542 (3)	0.8992 (15)	0.0306 (10)	0.034 (5)*	
C7	0.63057 (18)	0.73331 (10)	0.04389 (6)	0.0194 (2)	
O1	0.78156 (15)	0.43781 (8)	0.01487 (5)	0.02431 (19)	
O2	0.71931 (15)	0.52808 (8)	0.12494 (5)	0.0244 (2)	
O3	0.75288 (15)	0.54163 (8)	−0.11399 (5)	0.0253 (2)	
O4	0.53417 (16)	0.92717 (8)	−0.11604 (5)	0.0277 (2)	
H4C	0.545 (3)	0.9184 (17)	−0.1665 (10)	0.043 (5)*	
O5	0.61673 (15)	0.73672 (8)	0.12263 (5)	0.0243 (2)	
H5	0.643 (3)	0.6637 (17)	0.1401 (13)	0.057 (6)*	
N1	0.85916 (16)	0.24155 (9)	0.10129 (5)	0.01968 (19)	
H1	0.826 (3)	0.3064 (15)	0.0749 (11)	0.039 (5)*	
C8	0.91589 (18)	0.14661 (10)	0.06277 (6)	0.0194 (2)	
N2	0.97775 (16)	0.05353 (8)	0.10108 (5)	0.0199 (2)	
C9	0.98111 (18)	0.06016 (10)	0.17934 (6)	0.0191 (2)	
N3	0.92138 (17)	0.14924 (9)	0.22141 (5)	0.0222 (2)	
C10	0.86211 (19)	0.23957 (10)	0.18066 (6)	0.0205 (2)	
N4	0.8030 (2)	0.33205 (10)	0.21642 (6)	0.0274 (2)	
H4A	0.766 (3)	0.3971 (16)	0.1869 (11)	0.047 (6)*	
H4B	0.801 (3)	0.3291 (15)	0.2681 (9)	0.028 (4)*	
N5	0.90875 (19)	0.14847 (10)	−0.01381 (6)	0.0254 (2)	
H5A	0.855 (3)	0.2084 (14)	−0.0399 (10)	0.033 (5)*	
H5B	0.944 (3)	0.0848 (14)	−0.0407 (10)	0.032 (5)*	
N6	1.04817 (18)	−0.02974 (9)	0.21995 (6)	0.0235 (2)	
H6A	1.055 (3)	−0.0259 (16)	0.2725 (9)	0.033 (5)*	
H6B	1.102 (3)	−0.0896 (15)	0.1960 (10)	0.033 (5)*	
O1W	1.03866 (16)	0.57703 (9)	0.22527 (5)	0.0268 (2)	
H1A	1.108 (3)	0.5212 (19)	0.2061 (13)	0.059 (7)*	
H1B	0.925 (3)	0.573 (2)	0.2010 (14)	0.066 (7)*	
O2W	0.22325 (17)	0.76457 (9)	0.16102 (6)	0.0294 (2)	
H2A	0.171 (3)	0.7042 (18)	0.1850 (13)	0.059 (7)*	
H2B	0.349 (3)	0.753 (2)	0.1578 (14)	0.062 (7)*	

### Atomic displacement parameters (Å^2^)

**Table:**

	*U*^11^	*U*^22^	*U*^33^	*U*^12^	*U*^13^	*U*^23^
C1	0.0214 (6)	0.0183 (5)	0.0188 (4)	0.0010 (4)	−0.0005 (4)	0.0010 (4)
C2	0.0225 (6)	0.0170 (5)	0.0155 (4)	0.0011 (4)	0.0011 (4)	0.0009 (3)
C3	0.0220 (6)	0.0197 (5)	0.0164 (4)	0.0010 (4)	0.0010 (4)	−0.0020 (3)
C4	0.0257 (6)	0.0221 (5)	0.0154 (4)	0.0015 (4)	0.0011 (4)	0.0008 (4)
C5	0.0239 (6)	0.0192 (5)	0.0197 (5)	0.0016 (4)	0.0004 (4)	0.0022 (4)
C6	0.0281 (7)	0.0185 (5)	0.0183 (5)	0.0042 (4)	0.0019 (4)	0.0002 (4)
C7	0.0223 (6)	0.0197 (5)	0.0163 (4)	0.0015 (4)	0.0011 (4)	0.0007 (3)
O1	0.0338 (5)	0.0176 (4)	0.0215 (4)	0.0046 (3)	0.0016 (3)	0.0002 (3)
O2	0.0352 (6)	0.0205 (4)	0.0175 (4)	0.0044 (4)	0.0011 (3)	0.0024 (3)
O3	0.0389 (6)	0.0204 (4)	0.0168 (3)	0.0066 (4)	0.0026 (3)	−0.0019 (3)
O4	0.0423 (6)	0.0218 (4)	0.0193 (4)	0.0088 (4)	0.0020 (4)	0.0050 (3)
O5	0.0386 (6)	0.0197 (4)	0.0147 (3)	0.0064 (4)	0.0027 (3)	0.0006 (3)
N1	0.0254 (5)	0.0174 (4)	0.0163 (4)	0.0029 (4)	0.0014 (3)	0.0018 (3)
C8	0.0213 (6)	0.0197 (5)	0.0172 (4)	0.0006 (4)	0.0013 (4)	0.0007 (3)
N2	0.0251 (6)	0.0185 (4)	0.0161 (4)	0.0024 (4)	0.0012 (3)	0.0006 (3)
C9	0.0218 (6)	0.0179 (5)	0.0176 (4)	−0.0009 (4)	0.0004 (4)	0.0009 (3)
N3	0.0316 (6)	0.0189 (4)	0.0160 (4)	0.0038 (4)	0.0011 (4)	0.0003 (3)
C10	0.0242 (6)	0.0193 (5)	0.0179 (4)	−0.0001 (4)	0.0015 (4)	−0.0005 (4)
N4	0.0430 (7)	0.0214 (5)	0.0179 (4)	0.0079 (5)	0.0024 (4)	0.0000 (3)
N5	0.0363 (7)	0.0228 (5)	0.0170 (4)	0.0078 (4)	0.0014 (4)	0.0012 (3)
N6	0.0340 (7)	0.0186 (4)	0.0179 (4)	0.0042 (4)	0.0007 (4)	0.0024 (3)
O1W	0.0306 (6)	0.0269 (5)	0.0227 (4)	0.0023 (4)	0.0000 (4)	−0.0043 (3)
O2W	0.0324 (6)	0.0274 (5)	0.0286 (4)	0.0063 (4)	0.0024 (4)	0.0053 (4)

### Geometric parameters (Å, °)

**Table:**

C1—O1	1.2680 (14)	N1—H1	0.908 (16)
C1—O2	1.2772 (13)	C8—N5	1.3139 (15)
C1—C2	1.4768 (16)	C8—N2	1.3327 (15)
C2—C7	1.4120 (16)	N2—C9	1.3455 (14)
C2—C3	1.4124 (15)	C9—N6	1.3333 (15)
C3—O3	1.3580 (14)	C9—N3	1.3487 (15)
C3—C4	1.3865 (16)	N3—C10	1.3221 (15)
C4—C5	1.3967 (17)	C10—N4	1.3226 (16)
C4—H4	0.983 (15)	N4—H4A	0.942 (17)
C5—O4	1.3524 (14)	N4—H4B	0.890 (14)
C5—C6	1.3932 (16)	N5—H5A	0.902 (15)
C6—C7	1.3825 (16)	N5—H5B	0.917 (15)
C6—H6	0.975 (17)	N6—H6A	0.902 (15)
C7—O5	1.3622 (13)	N6—H6B	0.905 (15)
O3—H3	0.928 (19)	O1W—H1A	0.887 (19)
O4—H4C	0.880 (18)	O1W—H1B	0.88 (2)
O5—H5	0.921 (19)	O2W—H2A	0.906 (19)
N1—C10	1.3625 (14)	O2W—H2B	0.897 (19)
N1—C8	1.3642 (15)		
			
O1—C1—O2	122.41 (11)	C10—N1—H1	120.3 (12)
O1—C1—C2	119.33 (10)	C8—N1—H1	120.8 (12)
O2—C1—C2	118.26 (10)	N5—C8—N2	120.06 (11)
C7—C2—C3	117.01 (10)	N5—C8—N1	118.46 (11)
C7—C2—C1	121.90 (9)	N2—C8—N1	121.48 (10)
C3—C2—C1	121.05 (10)	C8—N2—C9	115.66 (10)
O3—C3—C4	118.48 (10)	N6—C9—N2	117.57 (11)
O3—C3—C2	119.91 (10)	N6—C9—N3	116.15 (10)
C4—C3—C2	121.60 (10)	N2—C9—N3	126.27 (11)
C3—C4—C5	119.17 (10)	C10—N3—C9	115.62 (9)
C3—C4—H4	119.0 (10)	N3—C10—N4	120.39 (10)
C5—C4—H4	121.8 (10)	N3—C10—N1	122.00 (11)
O4—C5—C6	116.43 (11)	N4—C10—N1	117.60 (11)
O4—C5—C4	122.40 (10)	C10—N4—H4A	119.7 (13)
C6—C5—C4	121.15 (11)	C10—N4—H4B	117.1 (12)
C7—C6—C5	118.77 (11)	H4A—N4—H4B	123.3 (17)
C7—C6—H6	119.4 (11)	C8—N5—H5A	120.1 (12)
C5—C6—H6	121.8 (11)	C8—N5—H5B	119.6 (11)
O5—C7—C6	117.08 (10)	H5A—N5—H5B	119.8 (16)
O5—C7—C2	120.62 (10)	C9—N6—H6A	118.7 (12)
C6—C7—C2	122.29 (10)	C9—N6—H6B	121.1 (12)
C3—O3—H3	103.2 (14)	H6A—N6—H6B	119.5 (17)
C5—O4—H4C	109.7 (13)	H1A—O1W—H1B	106 (2)
C7—O5—H5	105.9 (14)	H2A—O2W—H2B	109 (2)
C10—N1—C8	118.89 (10)		
			
O1—C1—C2—C7	178.44 (12)	C3—C2—C7—O5	179.37 (12)
O2—C1—C2—C7	−1.23 (19)	C1—C2—C7—O5	1.85 (19)
O1—C1—C2—C3	1.01 (19)	C3—C2—C7—C6	0.30 (19)
O2—C1—C2—C3	−178.65 (12)	C1—C2—C7—C6	−177.22 (12)
C7—C2—C3—O3	−179.54 (12)	C10—N1—C8—N5	178.60 (13)
C1—C2—C3—O3	−2.00 (19)	C10—N1—C8—N2	−1.51 (18)
C7—C2—C3—C4	−0.34 (19)	N5—C8—N2—C9	179.55 (12)
C1—C2—C3—C4	177.20 (12)	N1—C8—N2—C9	−0.35 (18)
O3—C3—C4—C5	178.81 (12)	C8—N2—C9—N6	−177.79 (12)
C2—C3—C4—C5	−0.4 (2)	C8—N2—C9—N3	2.79 (19)
C3—C4—C5—O4	−177.51 (13)	N6—C9—N3—C10	177.49 (12)
C3—C4—C5—C6	1.2 (2)	N2—C9—N3—C10	−3.09 (19)
O4—C5—C6—C7	177.54 (12)	C9—N3—C10—N4	−179.08 (13)
C4—C5—C6—C7	−1.3 (2)	C9—N3—C10—N1	0.93 (19)
C5—C6—C7—O5	−178.62 (12)	C8—N1—C10—N3	1.20 (19)
C5—C6—C7—C2	0.5 (2)	C8—N1—C10—N4	−178.79 (12)

### Hydrogen-bond geometry (Å, °)

**Table:**

*D*—H···*A*	*D*—H	H···*A*	*D*···*A*	*D*—H···*A*
O1W—H1B···O2	0.88 (2)	1.96 (2)	2.8015 (15)	160 (2)
O1W—H1A···O3^i^	0.89 (2)	2.04 (2)	2.8329 (13)	148 (2)
O2W—H2B···O5	0.90 (2)	2.01 (2)	2.8835 (16)	166 (2)
O2W—H2A···O1W^ii^	0.91 (2)	1.90 (2)	2.8050 (15)	173 (2)
N1—H1···O1	0.91 (2)	1.87 (2)	2.7729 (13)	174 (2)
N4—H4A···O2	0.94 (2)	1.89 (2)	2.8245 (14)	174 (2)
N4—H4B···O5^iii^	0.89 (1)	2.21 (2)	3.0049 (14)	148 (2)
N5—H5A···O2W^iv^	0.90 (2)	2.15 (2)	2.8319 (15)	132 (2)
N5—H5B···N2^v^	0.92 (2)	2.02 (2)	2.9339 (15)	179 (2)
N6—H6A···O3^vi^	0.90 (2)	2.33 (2)	3.1215 (14)	146 (2)
N6—H6B···O2W^vii^	0.91 (2)	2.01 (2)	2.9096 (15)	170 (2)
O3—H3···O1	0.93 (2)	1.64 (2)	2.5235 (12)	156 (2)
O4—H4C···O1W^viii^	0.88 (2)	1.86 (2)	2.7284 (13)	170 (2)
O5—H5···O2	0.92 (2)	1.70 (2)	2.5461 (13)	151 (2)
C6—H6···O4^ix^	0.98 (2)	2.58 (2)	3.5529 (15)	174 (2)

Symmetry codes: (i) −*x*+2, −*y*+1, −*z*; (ii) *x*−1, *y*, *z*; (iii) −*x*+3/2, *y*−1/2, −*z*+1/2; (iv) −*x*+1, −*y*+1, −*z*; (v) −*x*+2, −*y*, −*z*; (vi) *x*+1/2, −*y*+1/2, *z*+1/2; (vii) *x*+1, *y*−1, *z*; (viii) *x*−1/2, −*y*+3/2, *z*−1/2; (ix) −*x*+1, −*y*+2, −*z*.
